# Citrate-Linked Keto- and Aldo-Hexose Monosaccharide Cellulose Conjugates Demonstrate Selective Human Neutrophil Elastase-Lowering Activity in Cotton Dressings

**DOI:** 10.3390/jfb4020059

**Published:** 2013-05-17

**Authors:** Judson V. Edwards, Sonya Caston-Pierre

**Affiliations:** 1 USDA-ARS, Southern Regional Research Center, 1100 Robert E. Lee Blvd., New Orleans, LA 70124, USA; 2 Dillard University, 2601 Gentilly Boulevard, New Orleans, LA 70122, USA; E-Mail: scaston-pierre@dillard.edu

**Keywords:** human neutrophil elastase, monosaccharides, chronic wounds, carbohydrate-protein recognition, HPLC

## Abstract

Sequestration of harmful proteases as human neutrophil elastase (HNE) from the chronic wound environment is an important goal of wound dressing design and function. Monosaccharides attached to cellulose conjugates as ester-appended aldohexoses and ketohexoses were prepared on cotton gauze as monosccharide-citrate-cellulose-esters for HNE sequestration. The monosaccharide-cellulose analogs demonstrated selective binding when the derivatized cotton dressings were measured for sequestration of HNE. Each monosaccharide-cellulose conjugate was prepared as a cellulose citrate-linked monosaccharide ester on the cotton wound dressing, and assayed under wound exudate-mimicked conditions for elastase sequestration activity. A series of three aldohexose and four ketohexose ester cellulose conjugates were prepared on cotton gauze through citric acid-cellulose cross linking esterification. The monosaccharide portion of the conjugate was characterized by hydrolysis of the citrate-monosaccharide ester bond, and subsequent analysis of the free monosaccharide with high performance anion exchange chromatography. The ketohexose and aldohexose conjugate levels on cotton were quantified on cotton using chromatography and found to be present in milligram/gram amounts. The citrate-cellulose ester bonds were characterized with FTIR. Ketohexose-citrate-cellulose conjugates sequestered more elastase activity than aldohexose-citrate-cellulose conjugates. The monosaccharide cellulose conjugate families each gave distinctive profiles in elastase-lowering effects. Possible mechanisms of elastase binding to the monosaccharide-cellulose conjugates are discussed.

## 1. Introduction

Carbohydrate-based wound dressings have received increased attention in recent years for their occlusive [1,2,3,4] and functionally interactive properties [5,6]. Carbohydrate-based dressings for burn and chronic wounds [7,8,9] demonstrate numerous functional properties that correlate with wound healing. Recently demonstrated properties that provide interactive wound healing as polysaccharide-based fibers [10] are cellulose and cellulose composites [11,12,13], xerogels [14,15], charcoal cloth [16,17], alginates [18,19,20], chitosan [21,22,23] and hydrogels [24,25]. These dressings also afford properties of absorbency, ease of application and removal, bacterial and odor protection, fluid balance, occlusion, and elasticity.

Although the clinician has a plethora of occlusive dressings to choose from, which maintain a moist wound healing environment, a recent systematic review reported that all modern dressings had the same efficacy in healing as saline or paraffin gauze [26]. Thus, it may be inferred that there is potential to improve on cotton-based dressings as occlusive dressings, as has been previously noted [8]. Previously we have shown that cotton wound dressings can also be tailored with molecular recognition components to selectively remove human neutrophil elastase and matrix metalloproteases from wound fluid [27,28,29]. The proteases including human neutrophil elastase and matrix metalloprotease found in high concentration in chronic wounds create considerable growth factor and extracellular matrix protein destruction preventing the wound from healing [30]. The design of wound dressings that selectively sequester proteases like elastase from the chronic wound is couched in the concept that molecular features and properties of the protease can be used to tailor the molecular design of the wound dressing needed for selective sequestration of the protease. Thus, the protease size, overall charge, and mechanism for binding protease substrate in the active site may be employed to tailor the fiber design to more selectively bind the enzyme to the dressing in the presence of other wound proteins. Active wound dressings that have been designed to redress the biochemical imbalance of the chronic wound in this manner are composed of peptides [31,32], collagen/oxidized regenerated cellulose [12,13,33], derivatized cotton [27,34], hydrogels [35,36], alginate [19], and foams [37], all of which have a mechanism of action for protease neutralization or sequestration including negatively charged fibers and gels, protease substrate recognition, dressing bound protease inhibitors [19,38,39,40], and controlled release protease inhibitors [31,41].

Cellulose-based dressings have been manufactured and utilized for the last two centuries as a standard wound dressing in the care of both acute and chronic wounds. Although it is still used in much the same manner as originally conceived there have been some fiber modifications that have improved its quality and versatility in medical applications. We have adapted an esterification of cellulose with citrate-linked esters of monosaccharides to study the affinity-enhancing properties of modified cotton gauze to bind elastase [28]. The open chain ketone and aldehyde isomers of monosaccharides have electrophilic character that may enhance binding to the active site of elastase. We compare here the preparation and activities of two series of aldo- and keto- hexose citrate-cellulose conjugates.

## 2. Results and Discussion

### 2.1. Preparation and Analysis of Keto- and Aldo-Hexose Conjugates of Cellulose

#### 2.1.1. Preparation of Monosaccharide-Cellulose Conjugates

**Figure 1:**
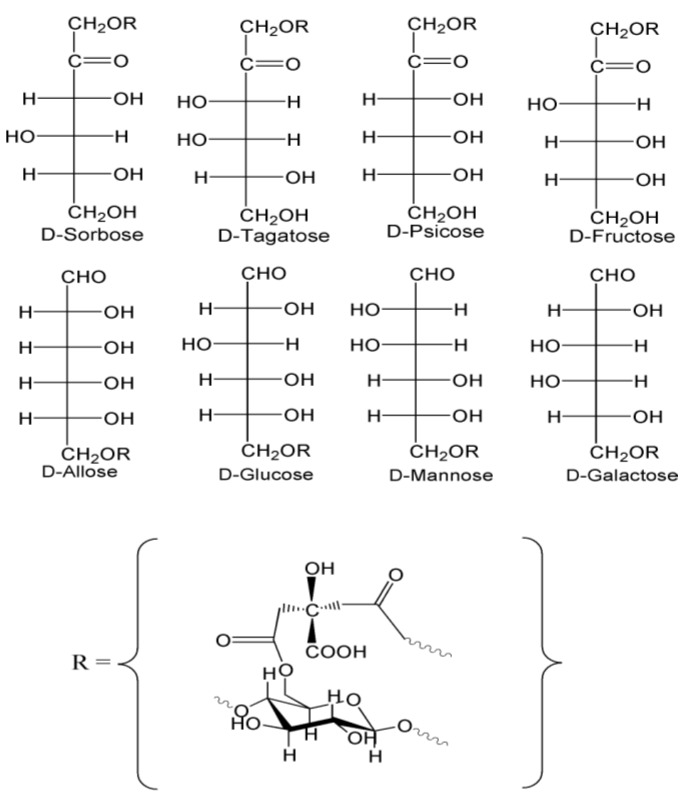
Structures for two pairs of aldohexose and ketohexose enantiomeric isomers conjugated to cellulose. The open chain reducing sugar is illustrated as the free aldehyde and ketone in equilibrium with the hemiacetal and hemiketal respectively. Below is the R group structure of one possible form of the substituted citrate-cellulose conjugate.

The keto- and aldo- hexose conjugates of cellulose were designed and prepared to test the comparative elastase binding effects of two different cellulose conjugate groups of monosaccharide isomers. The structural isomers of the two families of monosaccharides are shown in Figure 1. The configurational aldohexose isomers shown in Figure 1, which were conjugated to cellulose, are D-allose, D-mannose, and D-galactose. The ketohexose isomers which were conjugated to cellulose were D-sorbose, D-tagatose, D-psicose, and D-fructose. The open chain hemiacetal and hemiketal isomers illustrated in Figure 1 emphasize that the reducing sugars contain a free aldehyde and ketone functionality, respectively. The monosaccharides were linked to cotton cellulose with an acid-catalyzed citric acid reaction [28]. The structures in Figure 1 demonstrate the bonding in which the monosaccharide may be linked through the cellulose citrate ester.

#### 2.1.2. Characterization of Monosaccharide-Cellulose Conjugates

We have previously characterized the citrate linkage of cellulose-monosaccharide conjugates with both infra-red and HPLC analysis [28]. Characterization of the ester linkage in cotton cellulose with FTIR was first performed with photoacoustic spectroscopy [42]. In this study the citrate aldohexose and ketohexose conjugates of cellulose were characterized through base hydrolysis of the monosaccharide ester linked to cellulose followed by HPAE-PAD analysis of the hydrolysis products. Since the monosaccharides are attached to the cellulose fiber through an ester linkage to citrate which is in turn linked to cellulose, the citrate ester bond may be hydrolyzed by base treatment of the modified cotton gauze to give release of the monosaccharide. Release of the monosaccharide from its citrate ester linkage enables the analysis of the free monosaccharide. 

#### 2.1.3. HPLC Analysis of Monosaccharides

**Figure 2:**
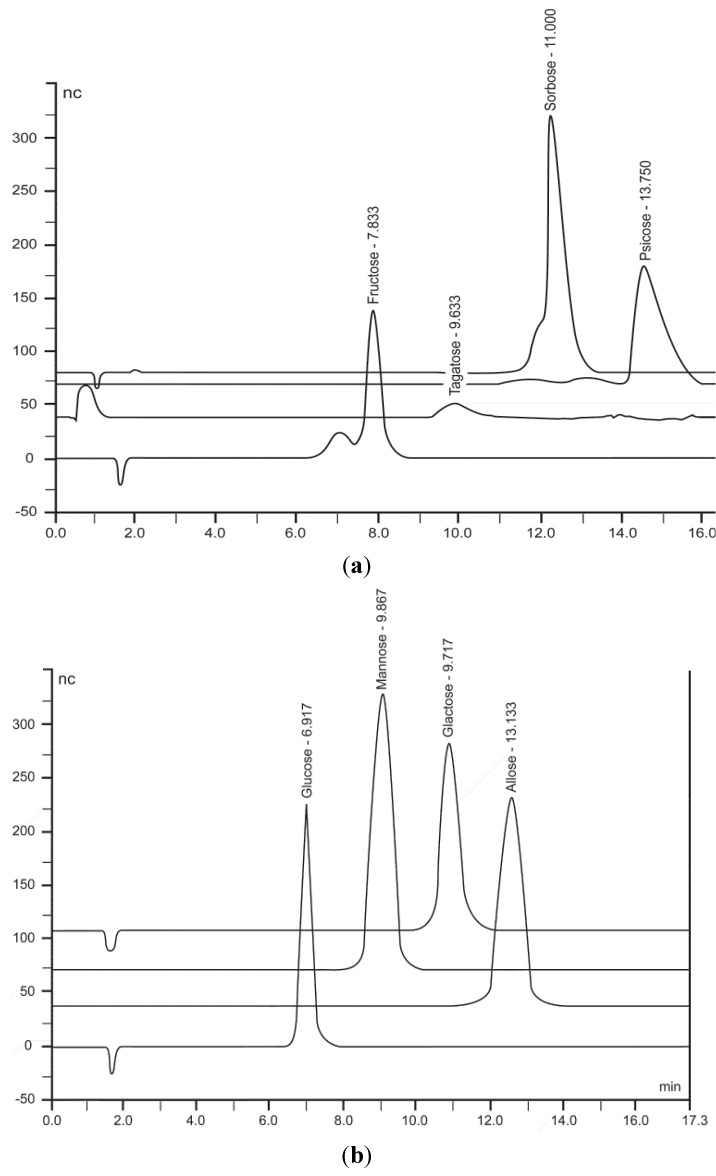
(**a**) HPLC chromatograms of ketohexoses released from cellulose conjugate; (**b**) HPLC chromatograms of aldohexoses released from cellulose conjugate.

The esterified monosaccharide released from the cotton fiber by base hydrolysis of the citrate ester was measured quantitatively using HPAE-PAD. The weakly acidic character of the monosaccharides allows partial ionization at the high pH of the chromatography eluant. The pulsed amperometric detection of the aldohexose and ketohexose is possible through measurement of the electrical current generated by their oxidation at the surface of a gold electrode. Figure 2 shows the elution profile of some of the monosaccharide samples injected onto the HPAE-PAD system for chromatographic analysis. Table 1 reports the monosaccharide levels found in the citrate-cellulose conjugates. The amounts of aldo- and keto-hexoses conjugated to cotton were found to be in a range of 1.20–4.12 milligram monosaccharide/grams.

**Table 1:** Quantitation of monosaccharides eluted from HPLC upon release from their cellulose ester conjugate.

Monosaccharides	Concentration of Aldo- & Keto-Hexose in ppms Determined in HPLC Eluant *	Levels of Monosaccharide Linked to Cotton Gauze **
Allose	405	4.0
Fructose	121	1.2
Mannose	210	2.1
Galactose	200	2.0
Glucose	419	4.1
Psicose	405	4.1
Sorbose	205	2.1
Tagatose	201	2.0

* Concentration of monosaccharides were determined through calibration of the monosaccharide in HPLC eluant; ** Levels of the monosaccharide are expressed as milligrams/gram of cotton based on the amount hydrolzyed from cotton samples as.

#### 2.1.4. FTIR Characterization of Cellulose Citrate Link

**Figure 3:**
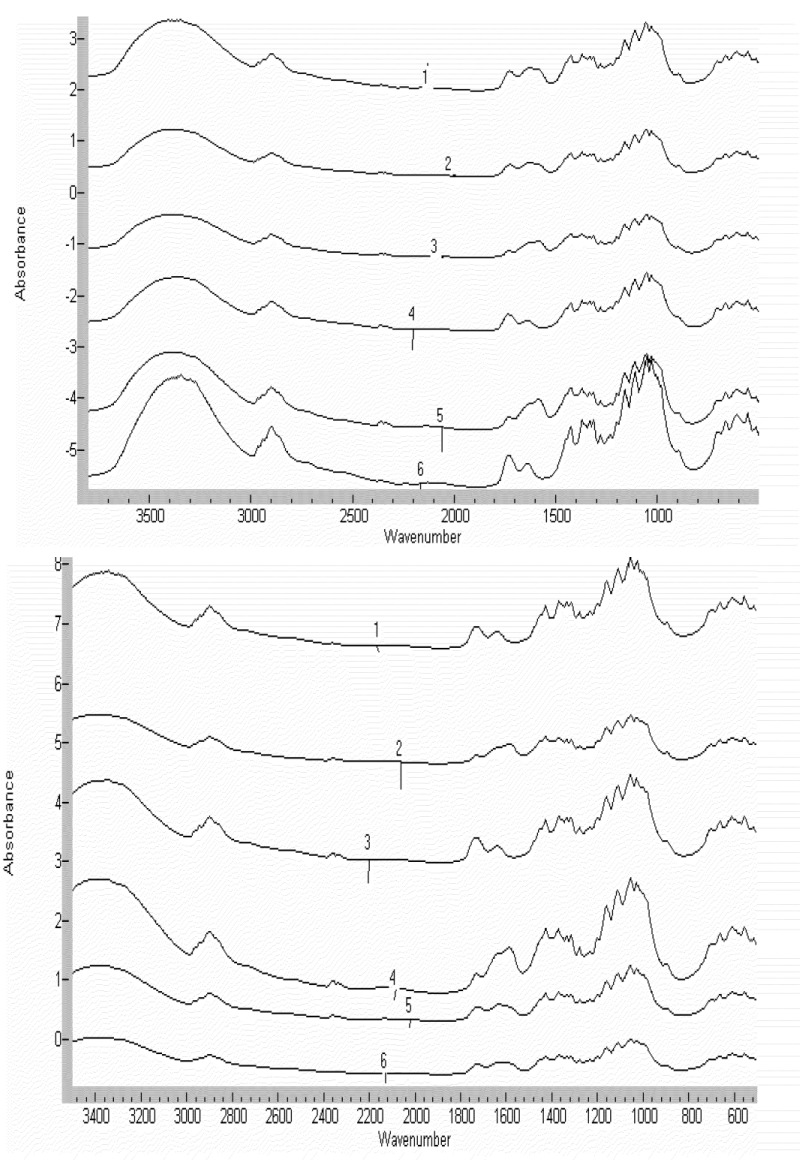
(**a**) FTIR spectra of representative ketohexose-citrate-crosslinked-cellulose cotton gauze: Fourier transform infrared spectra of crosslinked monosaccharide conjugates. (**1**) Spectrum of allose-cellulose crosslinked conjugate treated with 0.1 M HCl; (**2**) Spectrum of mannose-cellulose crosslinked conjugate treated with 0.1 M HCl; (**3**) Spectrum of allose-cellulose crosslinked conjugate treated with 0.1 M NaOH; (**4**) Spectrum of annose-cellulose crosslinked conjugate treated with 0.1 M NaOH; (**5**) Spectrum of allose-cellulose crosslinked conjugate; (**6**) Spectrum of mannose-cellulose crosslinked conjugate; (**b**) FTIR spectra of representative alohexose-citrate-crosslinked-cellulose cotton gauze. Fourier transform infrared spectra of crosslinked monosaccharide conjugates. (**1**) Spectrum of sorbose-cellulose crosslinked conjugate; (**2**) Spectrum of sorbose-cellulose crosslinked conjugate treated with 0.1 M HCl; (**3**) Spectrum of sorbose-cellulose crosslinked conjugate treated with 0.1 M NaOH; (**4**) Spectrum of tagatose-cellulose crosslinked conjugate; (**5**) Spectrum of tagatose-cellulose crosslinked conjugate treated with 0.1 M HCl; (**6**) Spectrum of tagatose-cellulose crosslinked conjugate treated with 0.1 M NaOH.

The cellulose analogs were also characterized by FTIR spectral analysis. By virtue of the citrate-linked cellulose the cotton fiber contains carbonyl groups that may be characterized as ester, carboxylic acid, and carboxylate anion functionalities. The citrate ester linkages in cotton cellulose can be distinguished from the corresponding citrate acid and carboxylate anions through IR analysis of acid and base treated fabric. In Figure 3 the FTIRs and the acid/base treatments of the cotton conjugates are shown. Each type of fabric containing the aldohexose and ketohexose conjugate was treated with 0.1M NaOH for 2 minutes at room temperature. Base hydrolyzes the ester bond between citrate and cellulose. Analogously the gauze is treated with 0.1 M HCl for 2 min at room temperature. Acid treatment protonates the free carboxylate anion. As seen in Figure 3 treatment of the citrate-linked fabric with base gives an increase in the intensity of the band at 1585 cm^−1^ and a decrease in the 1732 and 1735 cm^−1^ band intensity. This shift in carbonyl stretching band intensity corresponds to formation of the carboxylate anion. When the gauze is treated with 0.1 M HCl for 2 min at room temperature an increase in the band at 1725 cm^−1^ occurs and the bands at 1588 cm^−1^ and 1585 cm^−1^ disappear. This acid/base characterization of the citrate cellulose bond demonstrates the presence of the citrate linkage within the cotton fiber and is a corollary analysis to the HPLC-characterized monosaccharides released from the conjugates.

### 2.2. Human Neutrophil Elastase Sequestration by Modified Analogs

**Figure 4:**
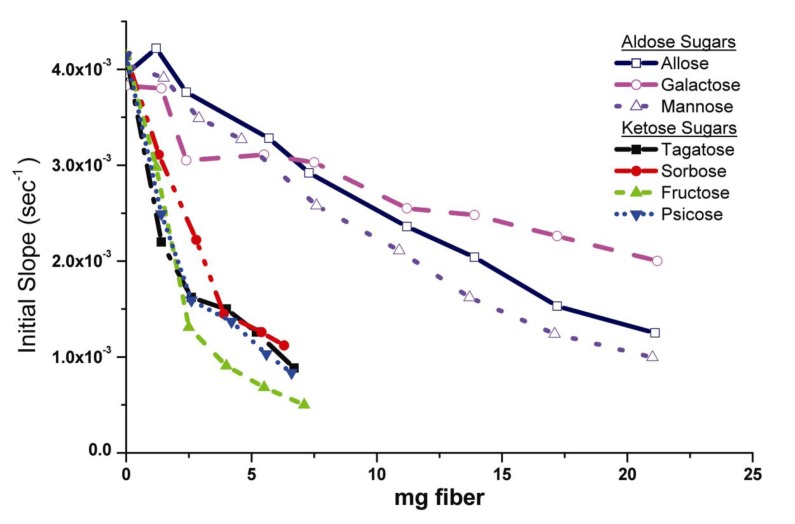
Sequestration binding of Human Neutrophil Elastase by Monosaccharide-Cellulose Conjuates on Cotton Gauze. Dose response curves plotted for both families of monosaccharides. Kinetic rate constants were obtained from reaction progress curves for each monosaccharide conjugate by assaying the conjugates of cellulose on cotton as described in the Materials and Methods section. The initial slope of each conjugate assay was plotted versus the weight of the cotton fiber used in the elastase uptake assay.

**Figure 5:**
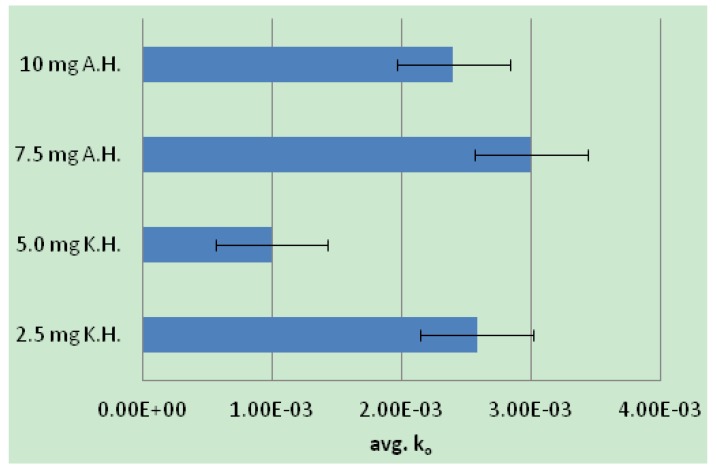
A bar graph comparing the initial rate constants (k_o_ sec^−1^)for keto- and aldohexose cellulose conjugates. A lower rate constant corresponds to higher elastase sequestration by the dressing. Initial rates are an average of three runs at each weight shown with standard deviations. A.H. = aldohexose, K.H. = ketohexose. Hence the lower rate at 5 and 2.5 mgs of the ketohexose conjugates reflect higher elastase sequestration.

The ability of the modified keto- and aldo-hexose conjugates to lower elastase activity in solution was measured by soaking the modified cotton gauzes containing the conjugates in solution and measuring enzyme activity remaining in solution following removal of the dressing. The dose response curves were plotted for both groups of monosaccharide-cellulose conjugates as shown in Figure 4. The dose response plots in Figure 4 are of the first order reaction rates taken from the reaction progress curves of elastase solutions, which were incubated with cotton fibers containing monosaccharide conjugate. Thus, elastase-lowering activity of the dressings is measured as a function of the elastase activity remaining in solution following incubation. In this manner the approach mimics removal of a dressing from the protease environment of the chronic wound. The activity profiles, which were based on determination of the initial rate constants, demonstrate a selective difference between the keto- and aldo- hexoses. The keto-hexose conjugates demonstrated a greater elastase-lowering activity than the aldohexoses. This difference in activity between the two families of monosaccharide conjugates is also apparent from the two distinct profiles of activity seen in Figure 4. Figure 5 shows the average rate constants based on elastase activity remaining in solution after incubation of the keto- and aldohexose crosslinked cotton dressings. The lower rates for the ketohexose analog also illustrate higher sequestration by the ketohexose appended dressings since less elastase is remaining in mimicked wound fluid.

#### 2.2.1. Mechanism of HNE Binding to Analogs

The different profiles of elastase uptake as seen for the keto- and aldo-hexose cellulose conjugates in Figure 4 prompted consideration of how enzyme, conjugate binding interactions that may be responsible for enhanced elastase binding to the conjugates may occur. Bimolecular interactions between proteins and carbohydrates are influenced by both polar and apolar surface interactions. Human neutrophil elastase is a glycoprotein and as such contains a significant carbohydrate portion (> 1000 amu) that has been shown to be glucosamine-based [43]. Hence the potential for interaction between the carbohydrate portion and the monosaccharide conjugate exists based on carbohydrate-carbohydrate interactions. Specific carbohydrate-protein interactions have been characterized with lectins for specific glucose and galactose recognition [44]. However, although protein carbohydrate interactions tend to be relatively weak *i.e*., binding constants for monosaccharides are in the range of 10^−3^–10^−4^ M [45], consideration of some precedents for bimolecular interactions between proteins and carbohydrates are worth examining as a corollary.

#### 2.2.2. Monosaccharide Carbonyl Binding

There are two types of carbonylic-based interactions that may occur, which could explain the preferred binding of the ketohexose versus the aldohexose observed. One possibility is that the monosaccharide conjugates undergo a Maillard-type reaction where the carbonyl carbon group of the monosaccharide and side chain amine groups, which are present in positively charged amino acids of the enzyme, react to form an imine. This type of interaction is more compelling as an explanation for binding because of the strongly basic nature of human neutrophil elastase. The imine-forming reaction between carbohydrates and proteins has been shown to occur with proteins in the body having long half lives [46], and may be at least partially responsible for the selective binding observed since ketohexose carbonyls would be expected to be more long-lived than aldohexose carbonyls, and thus more reactive with basic elastase side chains as a found in arginine and lysine. 

Another possible interaction is that the monosaccharide conjugates bind to the active site catalytic triad residue Serine-195, which is a selectively and uniquely reactive serine that is the central residue responsible for amide bond hydrolysis in serine proteases. The serine-195 hydroxyl side chain is made more nucleophilic by the adjacent histidine-57 and aspartate-102 through donation of the hydroxyl hydrogen to His-57 creating an anionic species. Thus, the Serine-195 hydroxyl’s enhanced nucleophilicity allows for interaction with an electrophilic functionality [47] as is the carbonyl carbon of the acyclic tautomer of a monosaccharide. 

Since aldohexose and ketohexose monosaccharides may alone bind weakly to the active site of elastase, it is hypothesized that the carboxylate functionality within the citrate-cellulose linker may facilitate binding through formation of a salt bridge with positively charged residues in the active site *i.e*., histidine or arginine and the aldo- or keto-monosaccharide from the citrate ester may interact analogous to the electrophilic substrate/inhibitor interaction found in chymotrypsin Ser-195. Examination of molecular models of monosaccharide-cellulose-citrate ester analogs that were allowed to dock in the vicinity of the active site of the enzyme demonstrated favorable binding of the open chain electrophilic carbonyl of the monosaccharide to the Ser-195. Thus, as viewed from the molecular modeling binding at the active site, it is energetically favorable and spatially accommodating for the ketohexose in its acyclic tautomerized form to undergo an electrophlic binding with the Ser-195 side chain hydroxyl [48].

#### 2.2.3. Elastase/Monosaccharide-Cellulose Subsite Binding Motifs

The putative sites in the conjuate that may enhance active site binding include (1) a hydrophobic site at the citrate fructose ester linkage; (2) a negatively charged binding site from a free citrate linker carboxylate; and (3) a hydrophilic binding site consisting of a hydroxyl group attached to the anomeric ketohexose ring. These proposed enzyme-binding sites are consistent with known substrate-based subsites characterized for the elastase active site binding [48,49]. For example the citrate carboxylate adjacent to the electrophilic carbonyl of the monosaccharide may contribute to the binding at the active site through an electrostatic interaction with a positively charged residue of elastase (Arg-217) which occupies the S3 subsite enzyme. There is precedent for this type of binding interaction with elastase in the inhibitor ursolic acid which also possesses a carboxyl negative charge that interacts with Arg-217 [50]. In addition the side chain hydroxyls of the threonine residues in a well characterized crystal structure [51] of porcine pancreatic elastase with the threonine hexapeptide serve as an indication that hydroxyl functionalities as those found in the anomeric pyranose and furanose rings are accommodated in the active site. This proposed mechanism of the monosaccharide conjugate’s enzyme active site binding is analogous to elastase inhibitor binding found in certain haloalkylketone- and aldehyde-based inhibitors, as previously reported [52]. The mechanism of binding in the catalytic triad of the enzyme active site by an elastase inhibitor acylation at the active site Ser-195 of the enzyme is also found with certain ketone inhibitors [53].

## 3. Experimental Section

### 3.1. Preparation of Crosslinked Cotton with Monosaccharide-Cellulose Conjugates

USP Type VII cotton gauze sponges (12 ply – 4 in. X 4 in.) were treated in solution pad baths consisting of 7% citric acid and 7% sodium hypophosphite and either 0.12 M of monosaccharides (all monosaccharides were obtained from Sigma Chemical Co., St. Louis, MO, USA. The gauzes were padded by two repetitions of dipping in the treatment solutions followed by removal of excess solution on a laboratory mangle with about 90% wet add-on. The padded fabrics were dried and cured at 155 ^o^C in ovens with mechanically circulated air. The treated gauzes were then washed under deionized water for one hour following the treatment. 

### 3.2. Chromatographic Analysis of Monosaccharides on Cotton Gauze

The monosaccharide conjugates of the cotton gauze were analyzed for glucose and fructose, and all of the analogous related ketohexose and aldohexose monosaccharides of this study by injecting hydrolyzed cellulose conjugate samples onto high performance anion exchange chromatography with pulsed amperometric detection (HPAE-PAD). Cotton gauze samples weighing approximately 800–900 milligrams that contain the cellulose citrate conjugates of the monosaccharide were soaked in 1 M NaOH for 30 min. The gauze samples were filtered and the eluant containing the monosaccahride was neutralized with 3N HCl to pH 7. All samples were filtered through a 0.45 m filter. The monosaccharide-treated samples were first diluted ten-fold. Monosaccharide concentrations in duplicate samples were determined on HPAE-PAD using a Dionex (Sunnyvale, CA, USA) BioLC instrument. Monosaccharides were separated on Dionex CarboPac PA-1 guard (25 × 4 mm) and analytical (250 × 4 mm) anion exchange columns, at a flow rate of 1.0 mL/min at ambient temperature (~25 °C). Column eluant conditions were: 16 mM NaOH isocratic (inject; 0.0–2.0 min), a gradient of 16–160 mM NaOH (2.0–26.0 min), followed by isocratic 200 mM NaOH (26.1–29.0 min), and return to 16 mM NaOH (29.1–32.0 min) to re-equilibriate the column with the initial mobile phase prior to the next sample injection. The monosaccharides (25 μL injections) were detected using integrated pulsed amperometric detection (IPAD). The PED-2 detector was equipped with Au working and Ag/AgCl reference electrodes, operating with the following working electrode pulse potentials and durations: E_1_ = +0.05 V (t_0_ = 0.00 s), E_2_ = 0.05 V (t_1_ = 0.42 s), E_3_ = +0.75 V (t_3_ = 0.43 s), E_4_ = +0.75 V (t_4_ = 0.60 s), E_5_ = −0.60 V (t_5_ = 0.61 s), E_6_ = − 0.60 V (t_6_ = 0.96 s). The duration of the IPAD integration interval was set at 0.2–0.4 s. Using a Spectra-Physics SP8880 autoinjector and Dionex Peaknet chromatography software, runs were accumulated of multiple samples and standards. Response factors were generated for each of the monosaccharides using check standards.

### 3.3. Fourier Transform Infrared Spectroscopic Measurements

A Nicolet Magna – IR 550 spectrometer was used for the FT-IR measurements. Resolution for all infrared spectra was 2 cm^−1^, and 250 scans for each spectrum. The finished cotton gauzes analyzed were ground in a Wiley mill to pass a 80 mesh screen. FT-IR spectra were taken of cotton powder samples prepared 5% by weight in potassium bromide pellets.

### 3.4. Enzyme Assay

Enzyme assays of the solutions containing unbound human neutrophil elastase were conducted in pH 7.6 buffer composed of 0.1 M sodium phosphate, 0.5 M NaCl, and 3.3% DMSO. Enzyme activity was measured by spectrophotometric monitoring of the release of p-nitroaniline at 410 nm from the enzymatic hydrolysis of the substrate N-methoxy-succinyl-Ala-Ala-Pro-Val-p-nitroanilide (MeO Suc-Ala-Ala-Pro-Val-pNA) (Sigma). The spectrophotometric kinetic assays were performed in a Bio-Rad Microplate Reader (Hercules, CA) with a 96-well format. Two hundred microliter aliquots of an elastase solution (0.2 units) were assayed per well, and 20 microliters of a 60 micromolar substrate solution was added to initiate the enzyme reaction.

Incubating each conjugated dressing in an elastase solution tested the lowering of elastase activity by monosaccharide citric acid cellulose conjugates of cotton gauze cellulose. Treated or untreated gauze samples were submerged in 1 mL of buffer containing 1 unit/mL of human neutrophil elastase. The samples were incubated for one hour at room temperature, after which each individual gauze sample was removed and placed in an Autovial press filter (Whatman) to extract unbound buffer and enzyme. The filtered fraction of each individual sample was re-combined with solution not taken up by the gauze and, the combined solutions were assayed for elastase activity as outlined in the assay above.

## 4. Conclusions

The putative role of the monosaccharide in the increased elastase binding of ketohexose-cellulose conjugate over the aldohexose, requires further work to examine monosaccharide-elastase interactions and elucidate the functionality responsible for selective monosaccharide-cellulose binding of elastase. However, this work corroborates earlier studies demonstrating the improved activity of fructose conjugates of cellulose (a ketohexose conjugate) over glucose conjugates and the increased sequestration activity of ketohexose over aldohexose conjugates in lowering elastase activity [28]. The study also sheds light on the potential to develop cellulose conjugate monosaccharides with selective bimolecular recognition and binding to elastase, and suggests a potential route to selective sequestration of destructive proteases in chronic wounds. 
